# Sexual satisfaction, an indicator of sexual health and well-being? Insights from STI/HIV prevention research in European men who have sex with men

**DOI:** 10.1136/bmjgh-2023-013285

**Published:** 2024-05-24

**Authors:** Karel Blondeel, Massimo Mirandola, Lorenzo Gios, Cinta Folch, Christiana Noestlinger, Maddalena Cordioli, Petra De Sutter, Marleen Temmerman, Igor Toskin

**Affiliations:** 1 Department of Sexual and Reproductive Health and Research (includes the UNDP/UNFPA/UNICEF/WHO/World Bank Special Programme of Research, Development and Research Training in Human Reproduction [HRP]), World Health Organization, Geneva, Switzerland; 2 Ghent University Faculty of Medicine and Health Sciences, Gent, Belgium; 3 Infectious Diseases Section, Department of Diagnostics and Public Health, University of Verona, Verona, Italy; 4 Centre Estudis Epidemiològics sobre les Infeccions de Transmissió Sexual i Sida de Catalunya (CEEISCAT) Departament de Salut, Generalitat de Catalunya, Barcelona, Spain; 5 Centro de Investigación Biomédica en Red de Epidemiología y Salud Pública (CIBERESP), Barcelona, Spain; 6 Institute of Tropical Medicine, Antwerpen, Belgium; 7 Centre of Excellence in Women, Adolescents and Child Health, The Aga Khan University, Nairobi, Kenya

**Keywords:** Cross-sectional survey, HIV, Prevention strategies, Public Health

## Abstract

**Introduction:**

Although sexual health has been holistically defined to include sexual satisfaction, it has been largely absent in health services and sexual and reproductive health and rights programmes in many parts of the world. We propose sexual satisfaction as a useful indicator, as one of the proxy measures for sexual health and well-being and as a component of well-being in general.

**Methods:**

The Sialon II project is a multicentre biological and behavioural cross-sectional community-based survey implemented across 13 European cities during 2013–2014 among men who have sex with men. Sexual satisfaction was explored using one single item: ‘How satisfied are you with your sex life?’ A multivariable multilevel logistic random-intercept model was estimated to identify factors associated with reporting positive sexual satisfaction versus negative sexual satisfaction.

**Results:**

Age, the number of partners and self-reported HIV status were not significantly associated with sexual satisfaction in the multivariate model. Participants reporting an insertive role or reported both an insertive and receptive role during the last anal intercourse were more likely to be sexually satisfied, compared with a receptive role. Participants reporting anal intercourse with a condom were more likely to be satisfied than those declaring no anal intercourse in the last 6 months, but no significant association was found compared with anal intercourse without condom. Knowledge of HIV-serostatus concordance with the last sexual partner was positively correlated with sexual satisfaction. Having had sexual intercourse with non-steady partners only in the last 6 months was negatively correlated. The more positive participants perceived their work/school, parents and friends/acquaintances’ attitudes towards gay or bisexual persons, the higher the odds they were satisfied with their sexual life.

**Conclusion:**

Using a single item on sexual satisfaction in a bio-behavioural study, our analysis has shown that it is associated with individual, interpersonal and social/structural factors and has proven its usefulness as a sexual health indicator among men who have sex with men.

**WHAT IS ALREADY KNOWN ON THIS TOPIC**

- Previous studies have evaluated sexual satisfaction in men who have sex with men, defining it in different ways and using different scales. This research revealed factors that are negatively or positively correlated with sexual satisfaction. It has, however, not been included in many (bio)behavioural studies relating to STI prevention as an indicator of sexual health.

**WHAT THIS STUDY ADDS**

- This study adds the insight that sexual satisfaction as a single-item question within a larger behavioural survey is correlated with personal, interpersonal and structural factors and is useful as an indicator of sexual health.

**HOW THIS STUDY MIGHT AFFECT RESEARCH, PRACTICE OR POLICY**

- From a sex-positive perspective, sexual satisfaction should be included in surveys and programmes that target STI and HIV prevention. This sexual satisfaction information and programmes might have a potential for behaviour change complementary to motivations related to the absence of disease and ill health.

## Introduction

The WHO’s working definition of sexual health includes ‘a positive and respectful approach to sexuality and sexual relationships, as well as the possibility of having pleasurable and safe sexual experiences, free of coercion, discrimination and violence’.^1^ The Guttmacher-Lancet Commission on sexual and reproductive health and rights (SRHR) has acknowledged that although sexual health was defined holistically to include sexual satisfaction, it is one of the aspects that have been largely absent both in health services and organised SRHR programmes in many parts of the world. The commission found the global health literature on sexual health and well-being to be sparse in general.^2^ This was echoed by the directors of WHO and UNFPA, expressing their support particularly for the recognition of counselling and care related to sexual function and satisfaction as essential sexual and reproductive health services, and urging governments to include these services within their programming and financing strategies for achieving universal health coverage.^3^ Both publications also called on the responsibility of governments to address the sexual and reproductive health needs of everyone, irrespective of their sexual orientation or gender identity.

While there is a sizeable literature on sexual satisfaction among male–female mixed-sex couples, research including sexual and gender minorities is relatively scarce and incoherent.^4 5^ Sexual satisfaction has been of interest to scientists working across a number of fields, including sexual function, relationship quality, quality of life in clinical settings and sexual health.^6^ Various concepts, theoretical models and measurement strategies have been used, resulting in findings that are often inconsistent and hardly commensurate.^7^

Sexual satisfaction has first been conceptualised in the context of sexual dysfunction and the frequency of sexual intercourse or orgasm. Later research has added affective and relational aspects, recognising both a physical and affective/emotional component to sexual satisfaction.^8^ Lay people’s definitions of sexual satisfaction highlight that it is ‘embedded in a net of relational (dyadic processes) and individual (personal sexual well-being) concepts’, moreover, the notion that men’s sexual satisfaction is predominantly predicted by physical and sexual aspects has been questioned.^9 10^ Aspects of sexual satisfaction might thus include among others genital health, psychological state, the quality of (an) intimate relationship(s) and sexual experience(s), including both concepts of physical and emotional satisfaction.^7 11^

Studies on sexual satisfaction have often focused on committed dyadic relationships and while research on sexual minority committed relationships suggests that sexual satisfaction rates are similar to those of heterosexual couples,^12 13^ sexual minorities might have lower expectations, lowering the threshold to be romantically or sexually satisfied, as in many settings, they are not allowed the same sexual rights in the political domain as heterosexual persons.^14 15^

Recently, research emphasis has shifted towards how social and political inequities affect what persons expect from their sexual lives, how they behave and evaluate their experiences.^7^ This is specifically relevant for sexual minorities, such as men who have sex with men (MSM), as for these populations, sexual activity is in most contexts morally unacceptable and sometimes dangerous and illegal.^16^ Distal minority stressors such as homophobia, rejection, and discrimination and proximal minority stressors, such as internalised homophobia and concealment, have been shown to be indirectly associated with poorer relationships with close peers, through higher levels of shame.^17^ In sexual minority youth, it has been evidenced, sexual-identity differences in depression and anxiety were mediated by differences in their relationship experiences and expectations.^18^ Internalised stigma has been shown to negatively affect the health status and relationships, including sexual, of gay men’s lives.^19–22^ The minority stress framework has exposed the relations between stigma and poor health among sexual and gender minorities.

More recently, using a sex-positive approach, research has proposed to instrumentalise sexual satisfaction as one of the indicators for, and an essential component of sexual health and well-being.^23–25^

Reviewing the literature, particularly on MSM, sexual satisfaction has been found to be associated with a wide array of factors, among others personal factors such as age, educational level, sexual compulsivity, depression, anxiety, stress, internalised homophobia or comfort with one’s homosexuality, alcohol use, use of anxiolytica, being concerned about sexually transmitted infections; or interpersonal factors such as satisfaction during the first sexual experience, not having a primary sexual partner, being partnered with a person living with HIV, relationship satisfaction and duration, frequency of sexual contact, sexual distance, the number of sexual behaviour communications, the inability to decline unwanted sex, partner support anal intercourse with(out) a condom; but also social and structural ones, such as experiences of HIV-related discrimination, history of having been intimidated or forced into unwanted sexual activities, homophobia or sexual stigma.^12 26–40^

In line with WHO’s comprehensive sexual health indicators and operational framework to monitor progress towards universal access to sexual and reproductive health, indications on what it means for MSM to have sexually satisfying lives can inform HIV/sexually transmitted infection (STI and sexual health interventions aiming to increase sexual health and well-being.^41–44^

We argue that sexual satisfaction, as one of the proxy measures for sexual health and well-being and a component of well-being in general, is a useful indicator. We hypothesise that among MSM enrolled in the Sialon II biobehavioural multisite survey, sexual satisfaction is negatively correlated with perceived stigma, and positively with self-disclosure of sexual orientation and the number of partners. We also investigated the relationship between sexual satisfaction and other personal and interpersonal variables on which the evidence is not conclusive.

## Methods

### Study setting

The Sialon II project is a multicentre biological and behavioural cross-sectional community-based survey implemented across 13 European cities during 2013–2014.

### Data collection

Two different sampling methods were used depending on the local context: time-location sampling (TLS) and respondent-driven sampling (RDS). Study procedures and biobehavioural data collection and testing methodologies have been described in detail elsewhere.^45^

### Study population

Men 18 years or older, having had sex (any kind of sex) with another man during the last 12 months, providing a consent form, agreeing to provide either an oral fluid specimen (in case of the TLS survey) or whole blood specimen (in case of the RDS survey) were included in the study.

### Participant and public involvement

Members of local MSM communities were involved in all aspects of the study, from its conception as well as during development, implementation (formative research, recruitment, data collection and prevention activities) and evaluation. They also participated in the interpretation and dissemination of the findings along with other stakeholders.^46^

### Instruments

A pen-and-paper questionnaire was designed based on the Global AIDS response progress reporting indicators guidelines and former relevant projects targeting MSM.^47 48^ Piloting and translation/back translation ensured consistency and quality of the items. The self-administered tool was used to gather information on demographic profile, sexual and testing behaviour, perceived stigma, and outness.

### Dependent variable

The dependent variable sexual satisfaction was explored using one single item: ‘How satisfied are you with your sex life?’ in both the TLS and RDS study arms. Possible answers were ‘very satisfied’, ‘somewhat satisfied’, ‘somewhat unsatisfied’, ‘very unsatisfied’ or ‘I prefer not to answer’. For data analysis, the item was dichotomised, combining the first two categories to satisfied and the following two to unsatisfied. Dichotomising satisfaction to reflect its presence or absence is consistent with other work on the subject.^49^ Participants who preferred not to answer were excluded from the analysis.

### Independent variables

#### Personal

Age (based on self-reported birth year), education (dichotomised as secondary school/high school or lower vs university degree or higher), the use of party drugs before or during last anal intercourse (ecstasy, cocaine, amphetamine, Gamma hydroxybutyrate (GHB), ketamine, mephedrone or crystal meth), self-reported HIV serostatus (positive, negative and unknown).

#### Interpersonal

Anal intercourse in the last 6 months (no intercourse, intercourse always with condoms or intercourse not always with condoms), the number of partners in the last 6 months (no partners, 1 partner, 2–3 partners, 4–10 partners and more than 10 partners), sexual role during last anal intercourse (versatile, receptive or insertive), assumed HIV serostatus concordance with last anal intercourse partner (concordant, discordant or uncertain) and relation to sex partners in the last 6 months (no partners, only steady partners, only non-steady partners or both steady and non-steady partners).

#### Social/structural

Perceived homopositivity, using a scale measuring respondents’ perceptions about ‘most people’s attitude towards gay or bisexual persons in the following contexts’: work/school, parents and friends/acquaintances. Each area was rated with a 5-point Likert scale, ranging from 1 (very negative) to 5 (very positive) (Cronbach’s alpha 0.73), the total varying from 3 to 15 points.

Outness was measured using the following question: ‘Thinking about all the people who know you (including family, friends and work or study colleagues), what proportion knows that you are attracted to men?’ Possible answers were ‘none’, ‘few’, ‘less than half’, ‘more than half’ or ‘all or almost all’. To facilitate the analysis, the variable was dichotomised, those out to none or few of the people they know were defined as not out; those out to less than half, more than half or all or almost all the people they know were defined as out.

### Data analysis

#### Descriptive and bivariate analysis

For quantitative variables, mean, median, SD, Wilcoxon-Mann-Whitney test and Kruskal-Wallis test by ranks were used. For nominal variables, we used percentages and Fisher’s exact test. Bivariate analysis was carried out using a logistic model and a p<0.05 was the threshold for including variables.

#### Multilevel modelling

A multivariable multilevel logistic random-intercept model was estimated; this allowed accounting for the hierarchical structure of the data collected by city and the consequent clustering of observations within study site.^50^ The multilevel analysis was conducted to identify factors associated with reporting positive sexual satisfaction versus negative sexual satisfaction. Predictors associated with the outcome variable with a p<0.05 were considered significant. STATA V.14.2 was used for all analyses (StataCorp). All variables were assessed for collinearity, and collinear variables were excluded from the final, adjusted models.

## Results

### Sialon II study sample

A total of 4901 MSM were enrolled across the 13 study sites. In Brussels, Sofia, Hamburg, Warsaw, Lisbon, Ljubljana, Barcelona, Stockholm and Brighton, 3596 participants were enrolled using TLS, in Bratislava, Bucharest, Verona and Vilnius, 1305 were enrolled using RDS. The characteristics of the sample have been described extensively elsewhere.^51 52^

In total, 3502 (77%) participants reported being satisfied with their sex life while 1065 (23%) reported being unsatisfied. In Brussels and Lisbon, participants reported the highest levels of sexual satisfaction, in Sofia the lowest (table 1).

**Table 1:** Sexual satisfaction—per city

City	No of observation	Satisfied with sex life (%)
Barcelona	386	82.9
Bratislava	369	75.8
Brighton	401	77.7
Brussels	381	82.9
Bucharest	158	84.6
Hamburg	393	76.2
Lisbon	398	85.2
Ljubljana	384	73.3
Sofia	410	60.2
Stockholm	345	74.6
Verona	372	75.6
Vilnius	298	77.8
Warsaw	385	71.1

### Sexual satisfaction: bivariate analysis

Results from the bivariate analysis are presented in table 2.

**Table 2:** Sexual satisfaction—bivariate analysis

	No of observations	Satisfied %	OR	95% CI	P value
Personal					
Age	4565		0.99	0.98 to 1.00	**0.00**
Education	4480				
High school or lower	1971	77.1	1		
Degree or higher	2509	76.4	0.91	0.78 to 1.05	0.19
Self-reported HIV status	4567				
Positive	256	82.4	1		
Negative	3442	77.3	0.83	0.60 to 1.17	0.29
Unknown	869	72.6	0.66	0.46 to 0.94	**0.02**
Use of party drugs last intercourse	4368				
Yes	403	79.2	1		
No	3965	76.7	0.90	0.69 to 1.17	0.43
Interpersonal					
Anal intercourse in the last 6 months	4567				
Anal intercourse with condom	1084	75.1	1		
No anal intercourse	802	70.6	0.71	0.56 to 0.88	**0.00**
Condomless anal intercourse	2681	79.2	1.40	1.18 to 1.67	**0.00**
Assumed HIV concordance last intercourse	3381				
Uncertain	1739	69.2	1		
Concordant	1483	84.0	2.25	1.89 to 2.69	**0.00**
Discordant	159	86.2	2.42	1.51 to 3.85	**0.00**
Number of partners in the last 6 months	4397				
1	862	81.4	1		
0	252	68.7	0.46	0.34 to 0.64	**0.00**
2–3	1039	75.1	0.68	0.55 to 0.86	**0.01**
4–5	630	79.1	0.87	0.67 to 1.13	0.29
6–10	747	71.4	0.63	0.50 to 0.81	**0.00**
>10	867	77.7	0.89	0.70 to 1.13	0.33
Sex role in the last 6 months	3919				
Receptive only	1346	71.8	1		
Insertive only	1415	79.7	1.45	1.21 to 1.74	**0.00**
Insertive and receptive	1158	79.3	1.45	1.20 to 1.75	**0.00**
Partnership in the last 6 months	3914				
Non-steady only	1168	61.1	1		
No partner	153	62.8	0.93	0.65 to 1.32	0.67
Steady only	581	89.2	4.93	3.69 to 6.60	**0.00**
Steady and non-steady	2012	82.3	2.89	2.44 to 3.42	**0.00**
Social/structural					
Perceived homopositivity	4566		1.10	1.06 to 1.13	**0.00**
Outness	4461				
Not out	1268	73.8	1		
Out	3193	77.7	1.15	0.98 to 1.35	0.08

P values in bold are <0.05, indicating the association between predictor and outcome variable reached the treshold for significance

In bivariate analysis, age, self-reported HIV status, type of anal intercourse, the number of partners, assumed HIV concordance, sex role, type of partnership and perceived homopositivity were significantly related to sexual satisfaction (p<0.05) and were selected for the multivariate model. The level of education and the use of party drugs before or during the latest anal intercourse were not. The results for outness seem to indicate that participants who were out about their sexual orientation were more likely to report being satisfied with their sexual lives, but the item did not reach statistical significance.

### Sexual satisfaction: multilevel multivariate model

Results from the multivariate model are shown in table 3.

**Table 3:** Sexual satisfaction—multilevel multivariate model

n=2913	OR	95% CI	P value
Age	0.99	0.98 to 1.00	0.23
Anal intercourse in the last 6 months			
Anal intercourse with condom	1		
No intercourse	0.61	0.42 to 0.88	**0.01**
Anal intercourse without condom	1.09	0.87 to 1.36	0.45
Assumed HIV concordance last intercourse			
Uncertain	1		
Concordant	1.52	1.23 to 1.87	**0.00**
Discordant	1.62	0.95 to 2.77	0.08
Sex role in the last 6 months			
Receptive only	1		
Insertive only	1.50	1.21 to 1.86	**0.00**
Insertive and receptive	1.35	1.07 to 1.71	**0.01**
Partnership in the last 6 months			
Non steady only	1		
No partner	0.75	0.40 to 1.41	0.38
Steady only	3.52	2.48 to 5.01	**0.00**
Steady and non-steady	2.53	2.04 to 3.13	**0.00**
Perceived homopositivity	1.10	1.06 to 1.15	**0.00**

P values in bold are <0.05, indicating the association between predictor and outcome variable reached the treshold for significance.

Not all predictors identified through the bivariate analyses were significantly associated with sexual satisfaction in the multivariate model. The number of partners was excluded from the model through variable selection, due to collinearity with partnership status. Age and self-reported HIV status did not reach significance (p>0.05). Outness almost reached significance in bivariate analysis and was, therefore, included in the first iterations of the multivariate model but did not reach significance either.

Participants reporting an insertive (colloquially named ‘top’) role or reported both an insertive and receptive (‘versatile’) role during the last anal intercourse were 1.50 and 1.35 times more likely to be sexually satisfied, in comparison to those reporting only a receptive (‘bottom’) role. Considering the type of sexual practice recorded in the last 6 months, participants reporting anal intercourse with a condom were more likely to be satisfied with their sexual life compared with those declaring no anal intercourse in the last 6 months (OR=0.61), compared with anal intercourse without condom, no significant association was found. Participants indicating they knew whether their HIV serostatus was the same or different from the serostatus of their last sexual partner had higher odds of being sexually satisfied than those that were uncertain (concordance: OR=1.52; discordance: OR=1.62; but not statistically significant, p=0.08). Having had sexual intercourse with steady partners only or with steady and non-steady partners in the last 6 months was associated with reporting satisfying sexual lives (OR=3.52; OR=2.53) compared with those reporting only non-steady partners. Lastly, the more positive participants perceived their work/school, parents and friends/acquaintances’ attitudes towards gay or bisexual persons, the higher the odds they were satisfied with their sexual life.

## Discussion

Over three-quarters of participants reported being satisfied with their sex life (77%). Our analysis seems to confirm that sexual satisfaction is regulated by personal, interpersonal and structural factors. Sexual satisfaction could thus potentially serve as an indicator of sexual health that looks further than the absence of sexual disease.

Increasing age did not predict decreased sexual satisfaction in our multivariate analysis. This is in line with other studies.^10 40^ Although increasing age has shown to come with less frequent sexual thoughts and activity, increased sexual dysfunction, and the presence of chronic diseases, other factors might become of greater importance in evaluating one’s satisfaction with sexual life, among other things greater intimacy with one’s partner(s) or changing attitudes towards sexuality.^53^ With regard to the attitude towards their sex life for example, Bourne *et al* 54 found among gay and bisexual men in 38 European countries, that although older men were less likely to describe their best sex life in terms of volume and variety, they were also less likely to refer to a relationship or emotional connection. Instead, they were more likely to specify their preferred sexual acts or behaviours.^54^ On the other hand, Fleishman *et al* have shown that among older people in same-sex relationships, relationship satisfaction was a significant predictor of sexual satisfaction.^55^

In our study, having had sex with steady partners only and both steady and non-steady partners, compared with having had non-steady partners only was positively correlated with sexual satisfaction while such correlation did not show for having had no sex partners. The desire for a relationship with another man and for some form of loving, intimate, or trusting connection with a sexual partner, is a frequently recurring theme in sex life for MSM.^21 54 56^ Having a steady sex partner could be an important way to gain confidence with one another, privacy and emotional support or to share positive events, whether that relationship is exclusive or not.^8^ Earlier research in gay male couples has shown that relationship satisfaction, sexual satisfaction, emotionally intimate experiences and sexually intimate experiences are positively related and other studies that sexual arrangements, either exclusive or nonexclusive, were not linked to sexual satisfaction.^12 37 57 58^ Other previous studies have also shown that for gay men the choice to live a monogamous relationship does not necessarily result in a more satisfying, functional and committed couple relationship.^59 60^ Hoff *et al* 57 have, however, shown that couples with discrepant agreements scored worse on relationship characteristics.^57^

Frequency of sexual intercourse has not been measured in our study, but the number of partners, although correlated in bivariate analysis, was not significantly correlated in multivariate analysis, indicating that having sex with a partner seems a bigger predicter for sexual satisfaction than the number of partners one had had sex with.

In line with other research, those MSM reporting a bottom sexual role, were less sexually satisfied. It has long been suggested that the bottom sexual role is symbolically related with a female gender role and the power culturally associated with it in many societies, where not being manly and fear of being vulnerable to another man, might be partly responsible for blocking the sexual response.^61^ Kiguwa^62^ showed that negative stereotypes, especially surrounding a ‘bottom’ identity, constrained sexual freedom and partner options, even though participants did not mention it as a reproduction of a traditional heteronormative gender script, but rather as an asset to control pleasure in the relationship or sexual encounter.^62^ A recent study on sexual minority individuals did, however, find a significant negative association between higher conformity to feminine norms and sexual satisfaction.^63^

In contrast with other research, outness was not correlated with sexual satisfaction.^54^ Gios *et al* have discussed the correlates of outness in this sample elsewhere.^64^ Self-reporting of outness might be affected by social desirability. The expected positive association between outness and sexual satisfaction might be reversed by higher external minority stressors.

Perceived homophobia was a negative predictor of sexual satisfaction. Homophobia manifests in the work, family and school environment, in the form of social exclusion, bullying and even violence.^65^ This form of prejudice is widespread in European societies, but less so in modernised, urbanised, post materialistically oriented countries with less religious influence.^19^ Earlier studies including models for sexual satisfaction showed a significant change by adding minority stress to the sociodemographic and sexual behaviour characteristics; those men who had a relatively negative opinion of their own sexual orientation reported lower levels of sexual satisfaction.^21^

Self-reported positive HIV status was not correlated with lower sexual satisfaction compared with negative HIV status in bivariate analysis. This correlation seems to have changed over time with the availability of better HIV-medication. Early research on MSM living with HIV did indeed observe loss of sexual interest (or enjoyment).^22^ It was hypothesised that sexual dissatisfaction among MSM in general might arise out of anxiety around being or becoming HIV infected, but those living with HIV might be especially affected by the divide between feelings of love or intimacy and sex without a condom on the one hand, and feelings of distance and sex with a condom on the other.^54 58 66 67^ People living with HIV are likely to face high levels of adversity related to multiple stigmatised identities.^37^ However, Prestage *et al* 68 found that men living with HIV were consistently more likely to agree that HIV treatments have improved the health outcomes for people living with HIV and reduced the likelihood of HIV transmission than were HIV-negative men.^68^ People living with HIV with undetectable viral load seem to score significantly higher on measures of satisfaction with their own health, pain and discomfort, sexual satisfaction, and concerns about the future.^69^ Our findings seem to confirm MSM living with HIV are not less satisfied with their sexual lives than those living without HIV. Although our study recruited participants before the availability of pre-exposure prophylaxis (PrEP, it is important to note that while studies have shown initiation of PrEP seemed to decrease sexual anxiety, it was not directly correlated with sexual satisfaction.^70 71^

An important finding from our study is that uncertainty about the seroconcordance of a sexual partner was associated with decreased satisfaction about the sexual life. Sexual intercourse between partners who do not know each other’s status may happen in circumstances where condom use negotiation is less evident or desirable, and when sexual partners know each other’s serostatus, the decision to have sex with or without a condom might be based less on a momentary or individual decision-making. Research on HIV serostatus knowledge and disclosure within the same Sialon II sample showed that study participants with undiagnosed HIV infection were significantly less likely to disclose their assumed negative serostatus to their most recent anal intercourse partner, leading the authors to the assumption that these participants have a lack of comfort with serostatus disclosure, increasing their exposure risk for HIV.^72^ This indicates that being uncertain about one’s HIV status and the incapacity of communicating about HIV concordance may negatively impact sexual satisfaction, as has been hypothesised in earlier research, but has not been researched extensively.^63^

### Limitations

There are various limitations to this study and generalisability of its results. It is important to mention that our study data were collected before the large-scale availability of PrEP, which dramatically changed the HIV landscape in Europe. This study used a single item to measure sexual satisfaction, as the settings of participant recruitment did not favour extensive surveys.^46^ Although Schwartz and Young rightly point out that using the term satisfied to measure satisfaction presumes that everyone knows what it means, while sexual satisfaction might mean different things to different people, this was part of the objectives of including this question in a biobehavioural survey.^73^ The study’s cross-sectional design precludes assessment of the causal direction of associations. Although the multilevel analysis takes into account the different cities, the sample comprises different populations against various social and political backgrounds, which have not been sufficiently assessed in this analysis. However, analyses per city showed similar, yet varying trends across all cities. Only 2913 of in total 4901 participants answered all questions providing data included in the multivariate analysis, which could have potentially biased the results. We have tested a model excluding the variable with the least available data, on assumed HIV concordance, including 3457 participants, and the correlations pointed in the same direction. Some of the variables included in the model have different time points, some ask for behaviour during the last 6 months while others for behaviour during the last anal intercourse, the latter may not represent an individual’s usual practice. The questionnaire did also not ask for gender identity.

## Conclusion

Using a single item on sexual satisfaction in a bio-behavioural study, our study has shown that it is associated with individual, interpersonal and social/structural factors and has proven its usefulness as a sexual health indicator that looks beyond the absence of disease and high-risk sexual behaviours. Understanding these associations offers an insight into the variety of determinants of sexual health and well-being for MSM and how to improve their overall well-being and quality of life. Including sexual health indicators such as sexual satisfaction has the potential to conceive interventions that are sex-positive and focused on rewards rather than focusing on the absence of negative aspects, sexual costs and risks.

Our study also points to the need of facilitating increased negotiation and communication skills, based on motivations concerning general well-being. Prevention of STIs focused solely on biomedical interventions may overlook what it means for individuals to have a satisfying sexual life. Alternatively, brief behavioural interventions between health providers and service users could focus on the motivation of the user to shift towards a healthier lifestyle while also creating the opportunity to offer interventions in areas traditionally outside of public health realm.^25 74^ Knowing and understanding the variety of factors that increase sexual satisfaction on an individual level, could potentially enable more effective responses to HIV and STI epidemics, by eliciting behaviour change, both increasing pleasure and decreasing harm.

## Data Availability

Data are available on reasonable request. Data are available on reasonable request, mail to massimo.mirandola@univr.it.
